# Role of the Dorsal Posterior Parietal Cortex in the Accurate Perception of Object Magnitude in Peripheral Vision

**DOI:** 10.1177/20416695211058476

**Published:** 2021-12-06

**Authors:** Tristan Jurkiewicz, Romeo Salemme, Caroline Froment, Laure Pisella

**Affiliations:** 57484 Centre de Recherche en Neurosciences de Lyon (CRNL), Université de Lyon, Bron, France; Hospices Civils de Lyon, Service de Neuro-ophtalmologie, Bron, France; Centre Ophtalmologique Vendôme, Ophtalmologie, Lyon, France; 57484 Centre de Recherche en Neurosciences de Lyon (CRNL), Université de Lyon, Bron, France; 57484 Centre de Recherche en Neurosciences de Lyon (CRNL), Université de Lyon, Bron, France; Hospices Civils de Lyon, Service de Neuro-ophtalmologie, Bron, France; 57484 Centre de Recherche en Neurosciences de Lyon (CRNL), Université de Lyon, Bron, France

**Keywords:** cortical magnification, dorsal visual stream, visual perception, size constancy

## Abstract

Following superior parietal lobule and intraparietal sulcus (SPL-IPS) damage, optic ataxia patients underestimate the distance of objects in the ataxic visual field such that they produce hypometric pointing errors. The metrics of these pointing errors relative to visual target eccentricity fit the cortical magnification of central vision. The SPL-IPS would therefore implement an active “peripheral magnification” to match the real metrics of the environment for accurate action. We further hypothesized that this active compensation of the central magnification by the SPL-IPS contributes to actual object’ size perception in peripheral vision. Three optic ataxia patients and 10 age-matched controls were assessed in comparing the thickness of two rectangles flashed simultaneously, one in central and another in peripheral vision. The bilateral optic ataxia patient exhibited exaggerated underestimation bias and uncertainty compared to the control group in both visual fields. The two unilateral optic ataxia patients exhibited a pathological asymmetry between visual fields: size perception performance was affected in their contralesional peripheral visual field compared to their healthy side. These results demonstrate that the SPL-IPS contributes to accurate size perception in peripheral vision.

## Introduction

The perceptual size of an object varies depending on where it is located in the field of view, not only in depth but also depending on visual target eccentricity (horizontal distance relative to gaze). This is a phenomenon called “the size eccentricity effect”. In healthy subjects, this perception is altered such that objects in the periphery are perceived as smaller than objects in central vision (Baldwin et al., 2016; Kirsch et al., 2020). This effect increases with the eccentricity of the target presented in peripheral vision (Baldwin et al., 2016). This phenomenon could have an attentional component: by using an index on the location of the peripheral object (like in a Posner task) this difference in size is no longer observed (Kirsch et al., 2020).

Several other neural mechanisms come into play in the perception of size: the increase in size of the receptive field in peripheral vision (Nakahara et al., 2006) but also the greater number of photoreceptors in the macula compared to the peripheral retina causing an overrepresentation of central vision in the visual cortex, also called *central magnification* Daniel and Whitteridge, 1961). This distortion of the representation of visual space is found in almost all structures of the visual cortex (Marino et al., 2008; Qiu et al., 2006). Several studies have highlighted that areas located in the dorsal posterior parietal cortex in monkeys (Galletti et al., 1999) and humans (Fattori, 2009; Pitzalis et al., 2015; Rossit et al., 2013) are an exception: in this region, the representation of peripheral visual field is almost restored relative to the central visual field. This is a region important for visuo-motor integration (Perenin et al., 1988).

The effects of neurological lesions of the SPL-IPS have been long described as leading to optic ataxia (Perenin et al., 1988). Movements directed to peripheral visual targets are imprecise, and most often deviated toward the side of the injured hemisphere that means movements were hypometric (Blangero et al., 2010; Rossetti et al., 2010; Rossetti et al., 2017; Vindras et al., 2016). In central vision (free-vision), few errors were observed (patient outcomes were similar to those of healthy subjects) (Rossetti et al., 2010). One interpretation is that patients with optic ataxia largely underestimate the distance of objects in the visual field contralateral to the lesion (the ataxic field), which results in pathological hypometry of pointing movements. These underestimations pathologically increase when the eccentricity of the target increases, with a pattern surprisingly systematic intra- and inter-subjects (Biotti et al., 2012; Blangero et al., 2010; Pisella et al., 2017; Vindras et al., 2016).

A modeling of these optic ataxia hypometric errors as a function of the eccentricity of the visual targets revealed a logarithmic compression of the peripheral vision, very similar to the equations modeling the central magnification in the visual areas (Vindras et al., 2016). It was therefore hypothesized that one role of the SPL-IPS, injured in optic ataxia, is to actively compensate for central magnification and represent more realistically peripheral vision in visual topographic (retinotopic) maps (Vindras et al., 2016). It appears indeed logically that a visuomotor interface should have access to accurate metrics of the visual space in order to subtend manual interaction with the objects of the environment. The restoration of the place of peripheral vision is not complete though, since healthy subjects still exhibit slight hypometric pointing errors increasing linearly with eccentricity (Vindras et al., 2016). Accurate retinotopic visual maps with peripheral “re-magnification” (Vindras et al., 2016) would not only subtend a reliable metric of distance relative to gaze (eccentricity) but also of the spatial extent (size) of the objects to reach and grasp. Conversely, SPL-IPS damage would not only impair object localization in peripheral vision but also size estimation, as already suggested in a recent study exploring size-weight illusion (Hassan et al., 2020).

We therefore set up a protocol to test whether object size estimation, like object distance, is also imprinted by a pathological compression of peripheral vision after SPL-IPS damage (probably due to uncompensated central magnification). More specifically, we tested perceptual estimation of rectangle thickness in peripheral relative to central vision in three patients with optic ataxia. Our hypothesis was that SPL-IPS lesion will lead to a perceptual underestimation and uncertainty of object’s size in the ataxic visual field.

Notably, this is a purely perceptual study in optic ataxia, a condition that has been classically associated with action-specific impairments in the context of the functional interpretation of the dorsal and ventral visual stream as a perception-action dissociation (Milner and Goodale, 2006, 2008). This very influential duplex theory was largely funded on the absence of purely perceptual impairment in optic ataxia following dorsal posterior parietal cortex damage. Demonstrating concomitant perceptual and motor deficits in these patients would put forward that dorsal visual stream impairment is specific to spatial uncertainty rather than action-specific.

## Material and Method

### Ethics Statement

Three patients with optic ataxia were recruited: CF (39 years), MO (29 years) and IG (49 years). Ten age-matched participants between the ages of 27 and 46 years (mean age = 38.1 ± 6.6 years) and without neurological history or psychiatric disorder also took part to this study. Informed written consent was collected from all participants in accordance with CPP Northwestern 1 046/2017 registration number 2017-A02562-51.

### Inclusion Criteria

All participants were right handed, and all had normal vision or vision corrected-to-normal using contact lenses. The age of recruitment was set between 25 and 50 years. Recruitment of appropriate control participants was not easy and interim statistical analysis was decided, at 10 participants with a reasonable power (zcc >= 2, power of statistical test of 70%) (McIntosh and Rittmo, 2021).

Optic ataxia patients were chronic, diagnosed with no primary visual and motor impairment (except right lower quadrantanopia for patient IG), and willing to participate in research protocols.

### Cases’ Summary

**Figure 1.**
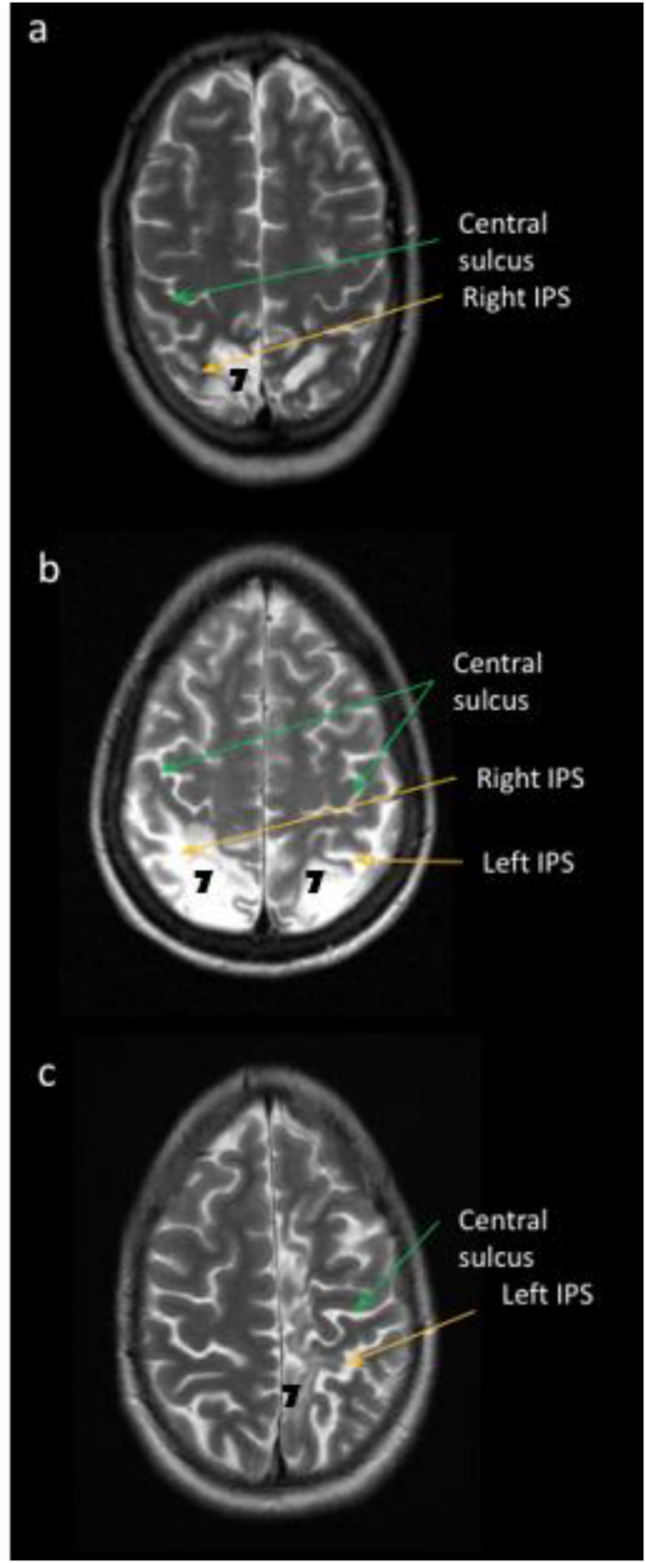
MRI horizontal T2-weighted images of optic ataxia patients. Patient CF (a), patient IG (b), and patient MO (c) display lesion of BA 7 (SPL-IPS region).

Patient CF suffered in 2003 from a posterior infarction of the junctional territories resulting in distributed and asymmetrical bilateral lesions of the Brodmann areas (BA) 2, 5, 7, 18 et 19 (Figure 1a) (Pisella et al., 2013). Post-stroke examinations revealed chronic unilateral optic ataxia in his left visual field (Mikula et al., 2021), which was a consequence of a greater lesion in the right hemisphere of BA 7corresponding in human to SPL-IPS region of the dorsal posterior parietal cortex.

Patient IG suffered in 1998 from ischemic stroke related to acute vasospastic angiopathy in the posterior cerebral arteries. The focal lesion concerned mainly the BA 7, 18, 19, and a limited portion of BA 39 in both hemispheres (Figure 1b) (Pisella et al., 2013). Post-stroke examinations revealed chronic bilateral optic ataxia (Mikula et al., 2021).

Patient MO came to see a neurologist in 2017 because of difficulties in learning to drive, keeping her direction on the road and monitoring her peripheral environment (especially on her right side). MRI showed a vascular lesion of the left parietal region, which probably occurred during infancy based on her reported history. The lesion concerned mainly BA 2, 3, 5, 7, and 19 in the left hemisphere (Figure 1c). Following examinations revealed unilateral optic ataxia in her right visual field complicated by a proprioceptive and motor impairment of the right upper (and lower) limb.

Note that the damage of the patients is different in BA 19 (superior occipital gyri, jointly to the SPL-IPS lesion in IG and MO versus left middle occipital gyrus, separated from the SPL-IPS lesion in CF) (Pisella et al., 2013) but the three patients display optic ataxia without clinical neglect according to a common damage to BA 7 corresponding in human to SPL-IPS region of the dorsal posterior parietal cortex.

### Apparatus and Task Design

Participants were seated on a height-adjustable chair with a chin-rest and an eye-tracking (Cambridge Research System) checking their ocular fixation throughout each trial. If a saccade was detected, the trial was aborted and displayed again at the end.

**Figure 2.**
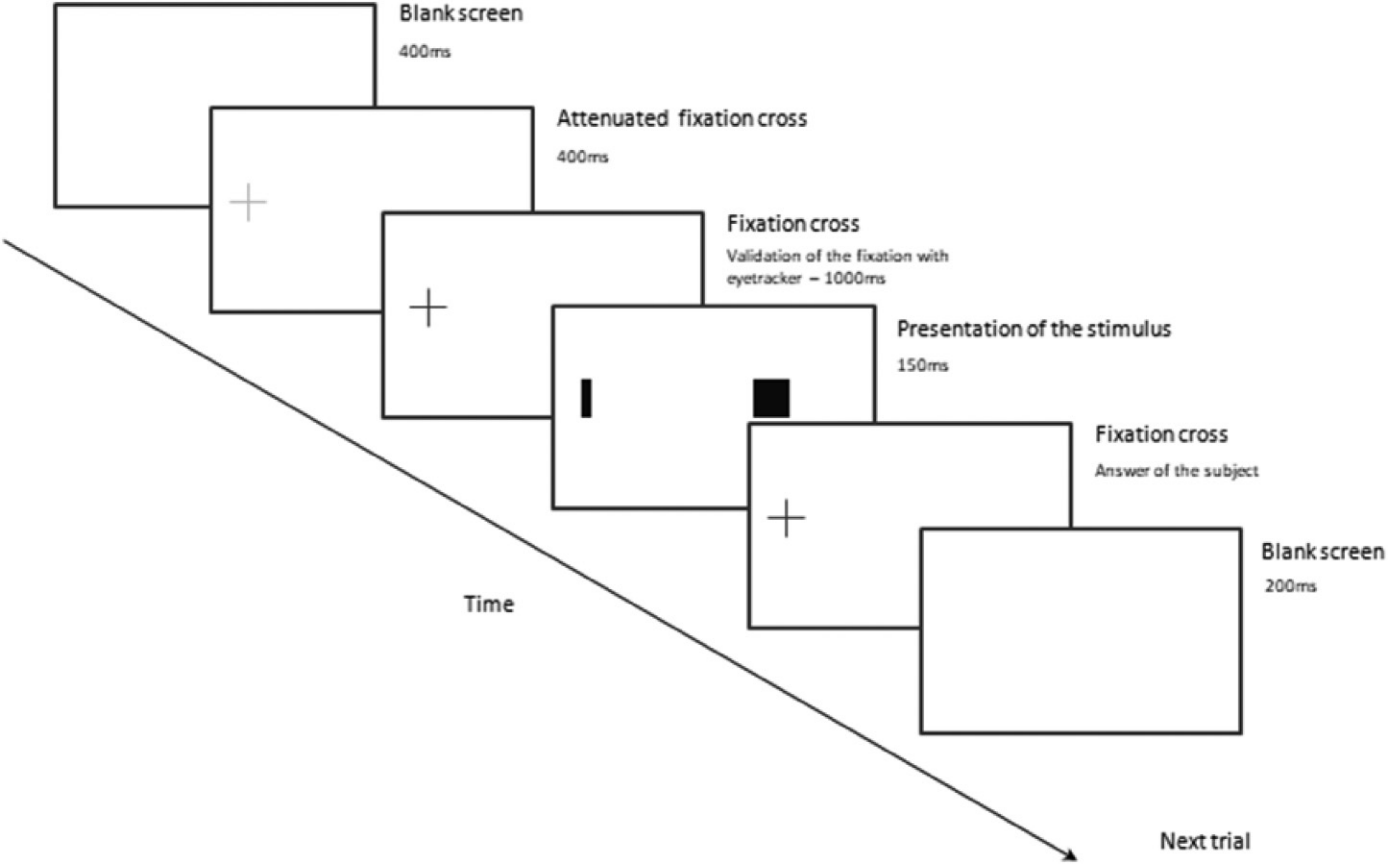
Experimental design. Example of the test of the right visual field (The fixing cross was central, the screen was shifted to the right to test the RVF). During the block, if the fixation is not validated by the eye tracker then the program will go on to the next one. The abandoned block will be randomly represented afterward.

The task consisted in comparing the thickness of two rectangles presented simultaneously, one in central vision (with a fixed thickness of 8 mm and a length of 16 mm) and another one at 15° of visual eccentricity in peripheral vision (with a thickness varying from 1 to 20 mm with steps of 1 mm and a length of 16 mm). Each pair of stimuli was randomly presented and repeated six times (120 trials on the whole for each participant in each visual field). Rectangles were flashed for 150 ms on a screen at a distance of 80 cm to the subject (Figure 2). For each trial, participants had to judge whether the peripheral rectangle was thicker or thinner than the central rectangle, and respond by pushing the lower button for thinner, the upper button for thicker. Examination was performed in the left and then in the right visual field for all participants.

### Data Analysis

Data analysis was performed per subject and per visual field. Button press data were transformed into accuracy percentages for each thickness difference (in mm) between the central and the peripheral rectangles and then modeled using a sigmoid function (nonlinear regression logit of Statistica V12.5 software) that allowed us to extract the subjective equivalence point (PSE), indicating the bias, and the slope, indicating the uncertainty, observed for the comparison task performance.

The PSE corresponds to the difference in thickness (in mm) between the central and peripheral target at which the participant considered the two rectangles as being of equivalent thickness. A positive PSE means that the peripheral target is perceived as smaller than the central target by the subject (underestimate). While a negative PSE that the peripheral target is perceived larger than the central target (overestimation). The PSE ratio between the right and left visual fields was computed in unilateral optic ataxia patients, a ratio close to 1 indicating low asymmetry.

The slope of the sigmoid curve allows us to assess the responses’ variability for each participant. A sharp slope indicates a possibly biased but precise differentiation of the size differences between central and peripheral vision. On the contrary, a shallow slope would be the signature of increased variability in the size estimation performance of the subjects.

### Statistics

Descriptive statistics (mean and standard deviation) and Wilcoxon's tests were performed for the group of healthy subjects to compare performance between the two visual fields using Statistica V12.5 software for Windows (StatSoft, Inc.). In order to test the hypothesis that the performance of each patient reveals in the controlesional visual field a pathological underestimation of the peripheral object’s size and a pathological perceptual size uncertainty, we used unilateral Crawford’s modified *t*-tests comparing the individual patients’ performance to the mean and standard deviation of the control group (Crawford and Garthwaite, 2002). This was done for the PSE and the slope of the sigmoid obtained in each visual field for the bilateral patient IG, and in unilateral optic ataxia patients for the slope in their ataxic visual field and for their PSE ratios between visual fields. Bonferroni corrections for multiple comparisons were implemented, statistical significance was therefore set at *p* < 0.025 for patient IG (4 comparisons) and *p* < 0.05 for unilateral patient (2 comparisons). To determine the statistical effect, we used the r for Wilcoxon’s tests and the *Zcc* for Crawford’s tests (Crawford et al., 2010).

## Results

### Controls

**Figure 3.**
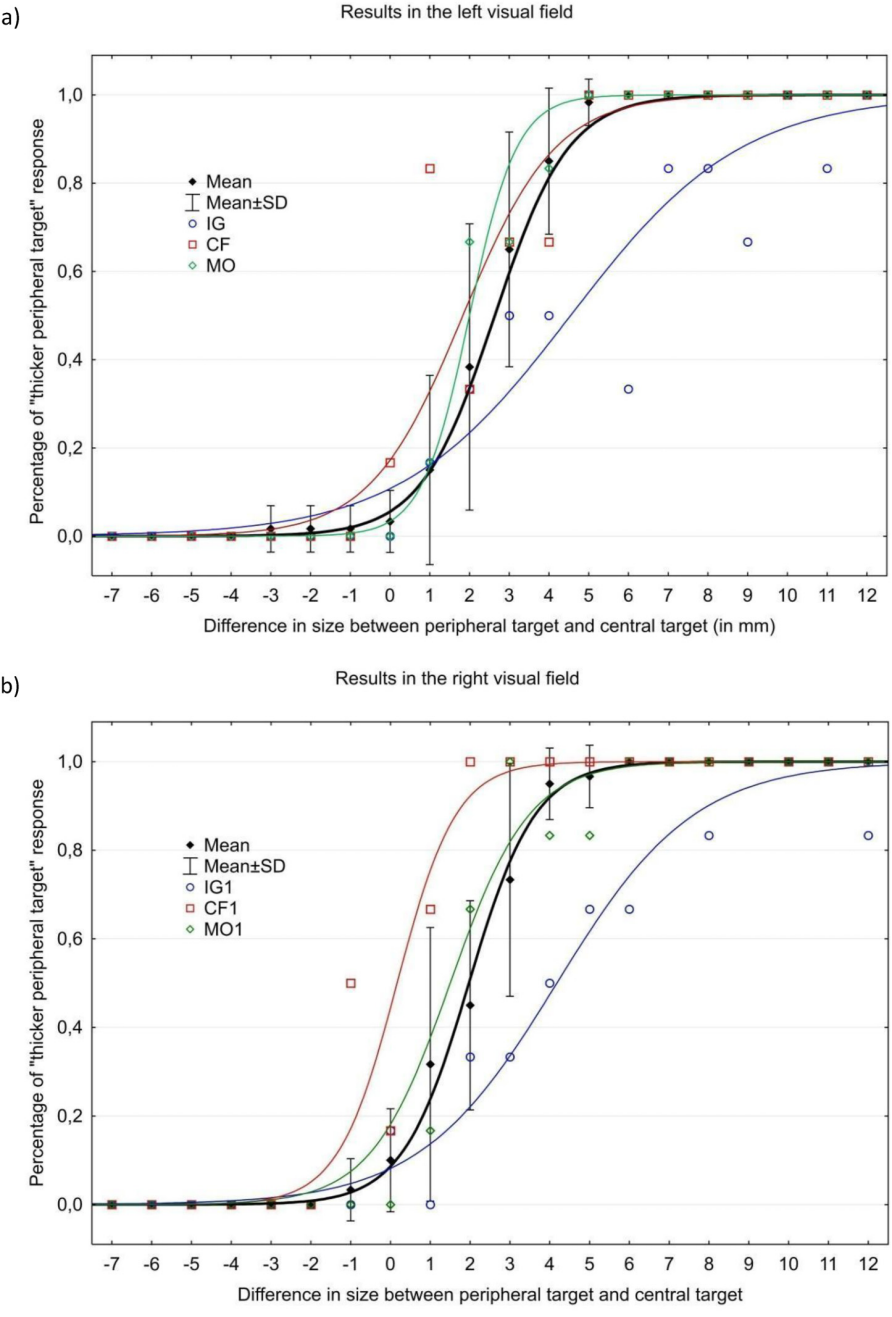
Results of size perception of subjects and patients in the left (a) and right (b) visuals fields. The full line represents the sigmoid modeling of the results of the different patients (IG in blue, CF in red, and MO in Green) and of the group of healthy subjects (in black). The slope of the curves represents the imprecision of the responses to the simulations (The less precise the responses during the task, the lower the slope of the curve). The PSE represents the difference in thickness between peripheral target and central target for which the targets are considered to be equivalent (50% response).

In controls, Wilcoxon's tests showed that there was no significant difference between the two visual fields for the PSE (*p* = 0.114, r = −0.354) and for the slope (*p* = 0.284, r = −0.251). PSE on average was 2.0 ± 0.88 mm in the right visual field (RVF) and 2.4 ± 0.7 mm in the left visual field (LVF) (Table 1) (Figure 3). This systematic positive bias confirms the “size eccentricity effect” (Baldwin et al., 2016; Kirsch et al., 2020) i.e. a stimulus in peripheral vision has to be at least 2 mm thicker to be perceived of the same size of a stimulus presented in central vision. The slope of the sigmoid curves was 0.370 ± 0.069 in the RVF and 0.422 ± 0.15 in the LVF on average in control subjects.

**Table 1.** Results of Control Group.

		RVF		LVF	
	Age	Mean PSE	Curve slope	Mean PSE	Curve slope
Subject 1	38	1.75	0.400	2.25	0.615
Subject 2	43	2.75	0.500	2.40	0.364
Subject 3	42	1.50	0.308	2.25	0.544
Subject 4	31	1.25	0.400	1.30	0.500
Subject 5	45	2.60	0.308	3.50	0.333
Subject 6	46	1.75	0.364	2.75	0.500
Subject 7	38	2.90	0.333	3.50	0.222
Subject 8	41	3.50	0.444	1.75	0.571
Subject 9	30	1.00	0.276	2.00	0.167
Subject 10	27	1.00	0.364	2.30	0.400
Mean	38.1	2.00	0.370	2.40	0.422
Ecart type	6.6	0.88	0.069	0.70	0.150

### Bilateral Patient

Patient IG who presents bilateral optic ataxia also exhibited a very similar PSE in left and right visual fields, 4,2 mm in the RVF and 4,5 mm in the LVF. The sigmoid fitting patient IG’s performance was clearly shallow, with a slope of 0.14 in the RVF and 0.114 in the LVF (Figure 3) (Table 2). The “size eccentricity effect” was pathologically increased in patient IG: the PSE was higher in the right (*p* = 0.02, *Zcc* = 2.50 (1.19 to 3.78)) and left (*p* = 0.009, *Zcc* = 3.00 (1.49 to 4.48)) visuals fields, as well as the slope in the right (*p* = 0.006, *Zcc* = − 3.33 ( − 4.96 to − 1.69)) visual field, compared to healthy subjects. The comparison for the slope in the left visual field did not reach significance after correction for multiple comparisons (*p* = 0.041, *Zcc* = − 2.05 ( − 3.16 to − 0.92)).

**Table 2.** Resume of Statistic Data and Effect Size.

			Significance test			Estimated effect size		Controls		
		*Cases’s score*		*t*	*p*	*point*	*95% CI*	*n*	*Mean*	*SD*
PSE	RVF	IG	4.2	2.38	0.02	2.5	(1.19 to 3.78)	10	2.00	0.88
		CF	0.2	−1.95	0.041	−2.045	(−3.15 to −0.91)			
		MO	1.6	−0.43	0.34	−0.455	(−1.10 to 0.21)			
	LVF	IG	4.5	2.86	0.009	3.0	(1.49 to 4.48)	10	2.40	0.7
		CF	1.85	−0.75	0.24	−0.79	(−1.49 to −0.054)			
		MO	2.0	−0.55	0.3	−0.57	(−1.23 to 0.11)			
	Ratio RVF/LVF	CF	9.25	14.47	<0.001	15.17	(8.28 to 22.08)	10	1.36	0.52
		MO	1.25	−0.2	0.42	−0.21	(−0.83 to 0.42)			
Slope of sigmoid curves	RVF	IG	0.14	−3.18	0.006	−3.33	(−4.96 to −1.69)	10	0.370	0.069
		CF	0.32	−0.69	0.25	−0.725	(−1.41 to −0.007)			
		MO	0.235	−1.87	0.047	−1.96	(−3.02 to −0.86)			
	LVF	IG	0.114	−1.96	0.041	−2.05	(−3.16 to −0.92)	10	0.422	0.15
		CF	0.216	−1.31	0.11	−1.37	(−2.24 to −0.48)			
		MO	0.40	−0.14	0.45	−0.147	(−0.77 to 0.48)			

### Unilateral Patients

Patient CF presenting left optic ataxia exhibited a PSE of 0.2 mm in the RVF and 1.85 mm in the LVF, while patient MO presenting right optic ataxia exhibited a PSE of 1.6 mm in the RVF and 2 mm in the LVF (Figure 3). PES’s ratio between visual fields was 9.25 for CF, 1.25 for MO, and 1.36 ± 0.52 for the control group. This represented a pathological asymmetry in CF (*p* < 0.001, *Zcc* = 15.17 (8.28 to 22.08)), but not for MO (*p* = 0.42, *Zcc* = − 0.21 (−0.83 to 0.42)).

For patient CF, the slope of the sigmoid curves was 0.320 in the RVF and 0.216 in the LVF, while for patient MO it was 0.235 in the RVF and 0.40 in the LVF (Figure 3). The slope was significantly shallower for patient MO in the RVF (*p* = 0.047, *Zcc* = − 1.96 (−3.02 to −0.86)) compared to the control group, but not for patient CF in the LVF (*p* = 0.111, *Zcc* = −1.37 (−2.24 to −0.48)).

## Discussion

The neural network and the neural mechanisms providing size constancy across space are still unknown. Besides, the IPS has been identified by neuroimaging studies as a neural substrate of magnitude processing, and Pinel, Piazza, Bihan, and Dehaene (2004) assumed a common code for the representation of symbolic (e.g., numerals) and nonsymbolic (e.g., numerosity, physical size, luminance) magnitude. Baldwin et al. (2016) and Kirsch et al. (2020) have described in healthy subjects the “size eccentricity effect” consisting in a slight under-estimation of the physical size of a target when it is presented in peripheral vision. This effect can be considered as an imperfect compensation of the cortical magnification of central vision by the brain. We found an exaggerated size-eccentricity effect in our optic ataxia patients following damage of the SPL-IPS region, suggesting its crucial contribution to the accuracy of magnitude perception over the visual field.

Three patients with optic ataxia and age-matched healthy subjects performed a task consisting in comparing the thickness of two rectangles flashed simultaneously, one in central and another in peripheral vision.

In healthy subjects, we observed with our paradigm an object size perceptual underestimation of approximately 2 mm at 15° of visual eccentricity with respect to central vision. These results confirm the small “size eccentricity effect” observed by the team of Baldwin et al. (2016) with another paradigm.

Following bilateral SPL-IPS lesion, patient IG showed a significant increase in the uncertainty of her responses and in the underestimation of the thickness of peripheral objects compared to healthy subjects, in both visual fields.

For patient MO, there was an increase in the uncertainty of responses in the ataxic field compared to controls. For patient CF the underestimation bias was significantly asymmetric between visual fields, larger in the ataxic field given the lack of peripheral under-estimation of the objects’ thickness in the healthy visual field. An important inter-hemispheric interaction is well-established at the level of the SPL-IPS regions in order to restore imbalance after unilateral stroke (e.g. Corbetta et al., 2005). This behavioral compensation may have provided to CF with a normal perceptual underestimation in the ataxic visual field thanks to a shift in response criteria.

These results can be read with a common interpretation along which patients with unilateral optic ataxia showed exaggerated visual field asymmetry of size perception at the expense of the ataxic (contralesional) field. Together with the results observed in bilateral optic ataxic patient IG, they therefore point toward a role of common damaged SPL-IPS region in perceptual size estimation in contralateral visual periphery. Size perception relies on a distributed neural network. A neuroimaging study (Konen and Kastner, 2008) found that visual coding in dorsal occipital area V3a and in parietal area V7 (IPS0) was size-dependent. These areas at the caudal part of the IPS (IPS0) and the parieto-occipital junction (POJ), like the parietal area V6a and the occipital area V6 (Fattori, 2009; Galletti et al., 1999; Pitzalis et al., 2015), display rare representations of visual space without a magnified representation of central vision, in contrast to more anterior zones in the intraparietal sulcus such as IPS1 and IPS2. In a recent paper (Vindras et al., 2016), we put forward the putative role of the dorsal posterior parietal cortex in the active “re-magnification” of peripheral vision.

Overall, note that the ventral visual stream (inferior occipital gyrus and inferior temporal cortex) is bilaterally preserved in all three patients. We therefore demonstrate that not only the ventral stream areas contribute to the visual perception, which challenges the duplex theory (Milner and Goodale, 2006; 2008) along which the ventral inferotemporal pathway is devoted to “vision for perception” and the dorsal occipito-parietal pathway to “vision for action”. This functional dissociation is questioned by the scientific literature, and dysfunction of attention mechanisms following SPL-IPS lesion may explain the visual perception deficits observed in optic ataxia (review in Pisella et al., 2021). Indeed, the attention spotlight can enhance visual processing in a specific part of the visual field that corresponds to a specific location in the retinotopic maps of the occipital cortex (Brefczynski et al., 2009; Brefczynski and DeYoe, 1999). Thus, at the location of attended targets in the occipital cortex, there is an enhancement of activity provoked by parietal cortex structures responsible of covert attention where central magnification is compensated. The size of this top-down visual cortex activation will be the same for attention toward an object located in peripheral and central visual fields. This is contrary to bottom-up activation of the visual cortex with central magnification, in which an object in the central visual field recruits more neurons during its activation compared to the same object in peripheral vision (Brefczynski et al., 2009). Lesion of SPL-IPS region will therefore cause the use of a visual representation map with a peripheral under-representation that explains the exaggerated “size eccentricity effect” in patients with optic ataxia. The small “size eccentricity effect” systematically observed in healthy subjects could therefore be due to the efficiency of the compensation of central magnification by the dorsal posterior parietal cortex (Vindras et al., 2016) possibly corresponding to attentional mechanisms known to improve spatial resolution (Brefczynski et al., 2009; Yeshurun et al., 1998).

## Conclusion

These results show how dorsal posterior parietal cortex contributes to accurate magnitude perception and judgment over the visual field.
